# Interfering antibodies may contribute to elevated d-dimer: a case report

**DOI:** 10.1186/s13256-024-04803-w

**Published:** 2024-09-29

**Authors:** Dorte B. Zilstorff, Thomas Steffen Hermann, Christine Rasmussen, Dorte Husum, Jørn Dalsgaard Nielsen, Nicolai J. Wewer Albrechtsen

**Affiliations:** 1 Department of Clinical Biochemistry, Copenhagen University Hospital-Bispebjerg and Frederiksberg Hospital, Copenhagen, Denmark; 2 Department of Cardiology, Copenhagen University Hospital-Bispebjerg and Frederiksberg Hospital, Copenhagen, Denmark

**Keywords:** d-dimer, Immunoassay, Interfering antibodies, Heterophilic antibodies, Case report

## Abstract

**Background:**

Plasma levels of d-dimer are elevated in patients with thromboembolisms. Here we investigated the existence of interfering antibodies as a potential cause for elevated d-dimer levels.

**Case presentation:**

A 42-year-old white Caucasian woman with a prior history of pulmonary embolism during her first pregnancy (treated with heparin therapy for 6 weeks postnatally) and hypothyroidism had a persistent elevated d-dimer without any clinical or ultrasound-based signs of thromboembolic conditions during her second pregnancy. We obtained informed consent and plasma was obtained from the patient. d-dimer levels were measured using two different assays. We also tested for the presence of rheumatoid factor, performed dilution series, and finally used an antibody depletion strategy. The two d-dimer assays performed similarly. Using our antibody depletion technique, we observed that ~ 1/3 of the increased plasma levels of d-dimer may be attributed to interfering antibodies.

**Conclusions:**

Our results identify interfering antibodies as a potential contributor to an increased d-dimer in this patient. Our case highlights the potential of heterophilic interference for increased d-dimer and provides a procedure to determine this analytically.

## Introduction

d-dimer is a fibrin degradation biproduct that is generated during the fibrinolysis of a blood clot [1]. Plasma concentration of d-dimer is commonly used in the diagnosis of venous thromboembolism (VTE), such as deep vein thrombosis (DVT) and pulmonary embolism (PE). However, d-dimer is an unspecific biomarker, as it is also elevated during malignancy, traumas, infection, postoperatively, and pregnancy [1]. d-dimer levels furthermore increase with age, and age-specific reference intervals are of great importance [1]. d-dimer levels must therefore be interpreted in a specific clinical context and combined with other diagnostic tools such as clinical risk assessment (Well’s DVT or PE score) and relevant imaging analyses [1].

Plasma concentration of d-dimer are most often measured with immune-based assays. Falsely elevated values may therefore theoretically be observed due to interfering heterophilic antibodies [2]. Antibody interference testing may include reanalyzing with other immunoassays or analytical principles, diluting samples, blocking interfering antibodies and removing interfering antibodies [2]. Heterophilic antibodies are most commonly reported to be of the immunoglobulin G (IgG), IgM, and IgA isotype [3]. Heterophilic antibodies as a cause of interference for d-dimer have previously been investigated in other case reports [4, 5]. However, none of them employed antibody depletion to investigate the direct effect of potential interfering antibodies.

### Key clinical question

We aimed to determine whether an elevated d-dimer without VTE is caused by interfering antibodies.

### Case history

**Fig. 1:**
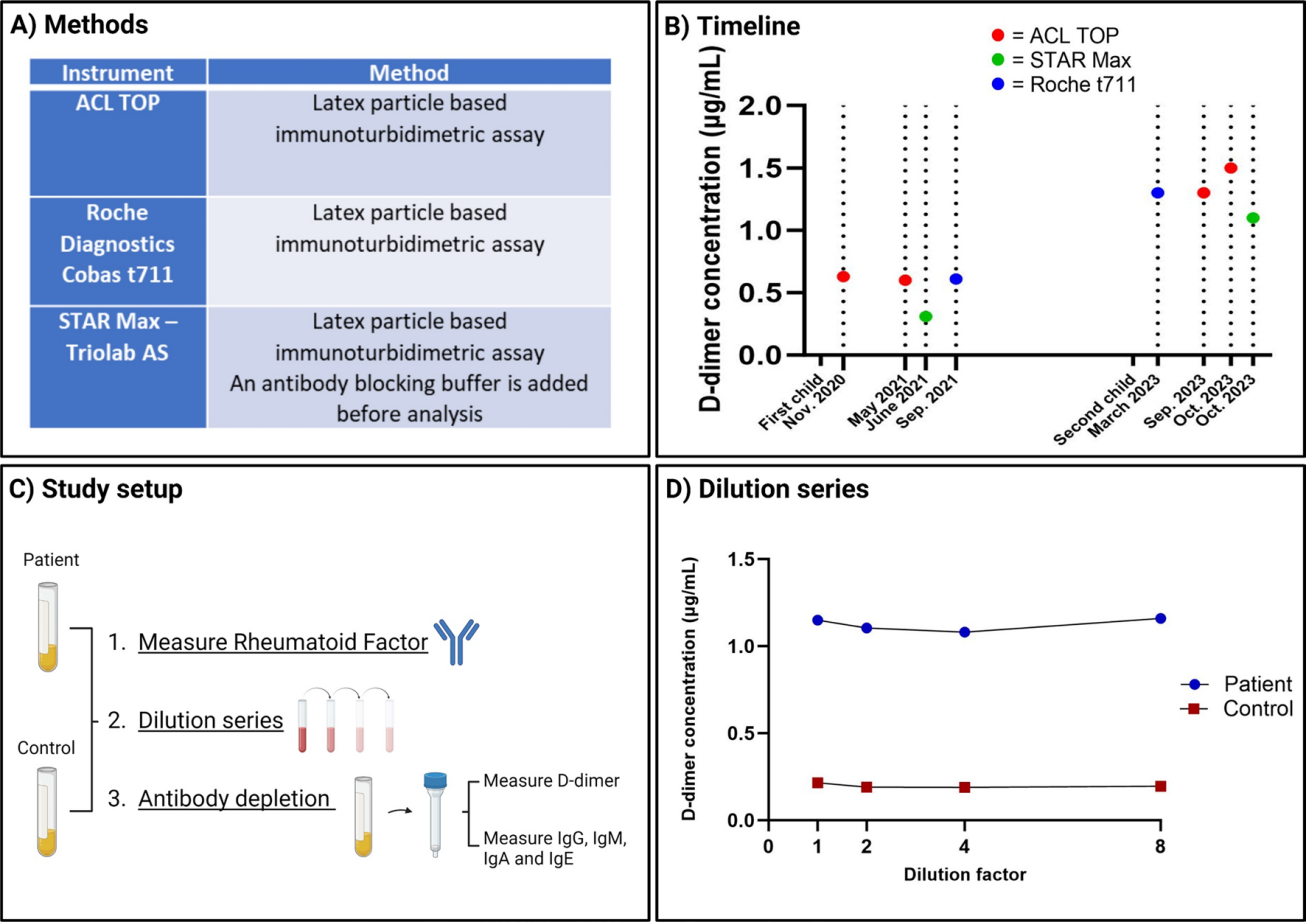
**A** Overview of the different instruments and methods used to analyze d-dimer in this case report. **B** Timeline for the measured d-dimer values on different instruments after first and second pregnancies, respectively. **C** Study setup. For both patient plasma and control plasma, rheumatoid factor was measured, dilution series were made, and antibody depletion was done before measuring d-dimer and antibodies. **D** Dilution series with the dilution factor ×1, ×2, ×4, and ×8 for both patient and control

Here, we investigated the potential interference of interfering antibodies as a cause of consistently elevated plasma d-dimer. The patient is a 42-year-old white Caucasian woman with factor V Leiden heterozygosity, with known Hashimoto’s hypothyroidism, who developed a PE during her first pregnancy in 2019/2020 treated with heparin 6 weeks postnatally. The patient’s hypothyroidism was stationary during the event and was treated with substitution. Following that event, d-dimer was consistently monitored for months using various immunoassays with results slightly above the reference value of 0.50 µg/mL, fluctuating between 0.6 µg/mL and 0.8 µg/mL, even though the patient had no signs of thrombosis, ultrasound verified (Fig. 1A, B). A single measurement of d-dimer concentration with STAR Max—Triolab AS, an assay including the addition of an antibody-blocking buffer, yielded a result within the reference interval (0.31 µg/mL), and therefore interfering antibodies were suspected (Fig. 1A, B). She had no subsequent thromboembolic event.

In late 2023, following the patient’s second pregnancy, d-dimer concentrations were again continuously elevated across different assays (1.3–1.5 µg/mL), still without any thromboembolic complications. Notably, an elevated d-dimer concentration was also measured when plasma was analyzed with the STAR Max—Triolab, yielding a result of 1.1 µg/mL (Fig. 1A, B).

Thus, we decided to investigate in depth whether this patient’s elevated d-dimer could be attributed to interfering heterophilic antibodies.

### Laboratory approach and discussion

To address this, we sampled blood using K_3_ ethylene diamine tetraacetic acid (EDTA) plasma tubes (Greiner Bio-one, Frickenhausen, Germany) and citrate plasma tubes (Greiner Bio-one, Frickenhausen, Germany) from the patient and a healthy control (negative control). Samples were centrifuged immediately, and plasma was stored for −20 °C until analysis.

Plasma was then analyzed for rheumatoid factor as rheumatoid factors, such as heterophilic antibodies, are well known to interfere with immunoassays [2]. Rheumatoid factor was analyzed on Roche Diagnostics—Cobas 8000 by an immunoturbidimetric assay. Both patient and control were negative for rheumatoid factor (< 14 × 10^3^ IU/L) (Fig. 1C).

We then performed serial dilutions and measured d-dimer on Roche Diagnostics—Cobas t711, respectively, for non-diluted and diluted ×2, ×4, and ×8. The dilution approach is commonly used to assess antibody interference but may be highly dependent on the affinity of the antibody. When corrected for the dilution factor, and the dilution series were linear for both control and patient. These data suggested no interfering antibodies (Fig. 1D).

Finally, we depleted both plasma samples (patient and control) for antibodies and albumin with High-Select™ HAS/Immunoglobulin Depletion Resin Midi Columns (cat. no. A36367, lot no. YJ382292) from ThermoFisher (Fig. 1C). To control for the dilution caused by the procedure itself, we also diluted the patient and control samples separately, using identical dilution factor (1:11) but without performing immunodepletion. Technical replicates were analyzed four times for the patient sample and two times for the control sample.

To access the efficiency of the antibody depletion, the samples were analyzed for IgG, IgM, IgE, and IgA on Roche Diagnostics—Cobas 8000. The antibody depleting kit successfully depleted 99 ± 1% (mean ± SD) of IgG, 89 ± 1% of IgM, 89 ± 13% of IgA, and 22 ± 13% of IgE from the patient’s plasma. For comparison, 100 + 0% of IgG, 95 + 1% of IgM, 99 + 0% of IGA, and 54 + 1% of IgE in the control plasma was successfully depleted.

The antibody-depleted samples and the non-depleted samples were then analyzed for d-dimer on Roche Diagnostics—Cobas t711. The mean values of the analyzed d-dimer concentrations are presented in Table 1.

**Table 1:** d-dimer concentrations

	Dilution factor	d-dimer mean ± SD (µg/mL)
Patient non-diluted	1:1	1.18
Patient antibody depleted	1:11	0.073 ± 0.021
Patient non-antibody-depleted	1:11	0.145 ± 0.068
Control non-diluted	1:1	0.192
Control antibody-depleted	1:11	0.056 ± 0.01
Control non-antibody-depleted	1:11	0.033 ± 0.013

Table 1d-dimer was analyzed in the non-diluted patient and control plasma. After antibody depletion or only dilution (1:11), d-dimer was analyzed in the patient and control plasma, and four technical replications were done for the patient sample (one outlier excluded for analysis) and two technical replications were done for the control sample.

We then calculated the ratio between the antibody-depleted sample and the non-depleted sample to estimate the potential interference of an inherent patient antibody. The antibody-depleted sample is 50% lower than the non-depleted sample, indicating that part of the elevated d-dimer is caused by heterophilic antibodies.

To determine the clinical impact of the inference caused by heterophilic antibodies, we calculated the percentage difference from the non-diluted sample and found that 32% of the elevated d-dimer in the patient sample can be explained by immunological interference.

As we chose a negative control in this case report, the same calculations cannot be done with the control d-dimer values, as we believe there is too high of analytical variance in the measured range when a normal low d-dimer is diluted with 11. For further studies we would analyze a positive control with a truly high d-dimer for comparison.

This case has several limitations, such as the potential analytical variance in the measured range for samples was diluted by a factor 11, however, this is unlikely to affect the patient–control ratio and the depleted–non-depleted ratio. The relative inefficacy of IgE depletion may lower the actual contribution of the interfering antibody(ies). On the contrary, IgE antibodies have not been reported to cause interference with immunoassays. The patient had a prior thromboembolic event, and it is well known that d-dimer is increased during pregnancy. We therefore cannot rule out that these factors contribute to the numerically elevated d-dimer reported here. Treatment of thromboembolic events, as was the case for this patient, may also cause consistently increased d-dimer. However, as shown by both assay dependency and depletion, the causation for the concomitantly increased d-dimer may more likely relate to heterophilic antibodies, but we cannot exclude contribution from prior treatment. Increased d-dimer has been reported in patients with factor V Leiden [6] mutation, however, as the patient had a normal d-dimer before her first pregnancy, we do not find it likely that the elevation in d-dimer levels are causally linked to this.

## Conclusions

Our results identify interfering antibodies as a potential contributor to increased d-dimer in a patient suspected for a thrombogenic event. The patient most likely does, however, have increased d-dimer independent of the contributing interfering antibodies, although the causation of this is currently unclear.

We believe our case may highlight the potential of heterophilic interference for increased d-dimer and provides a procedure to determine this analytically.

## Data Availability

All data are available on reasonable request to the corresponding author.
